# Nonconservative current-induced forces: A physical interpretation

**DOI:** 10.3762/bjnano.2.79

**Published:** 2011-10-27

**Authors:** Tchavdar N Todorov, Daniel Dundas, Anthony T Paxton, Andrew P Horsfield

**Affiliations:** 1 Atomistic Simulation Centre, School of Mathematics and Physics, Queen’s University Belfast, Belfast BT7 1NN, UK; 2 Department of Materials, Imperial College, London SW7 2AZ, UK

**Keywords:** atomic-scale conductors, current-induced forces, failure mechanisms, nanomotors

## Abstract

We give a physical interpretation of the recently demonstrated nonconservative nature of interatomic forces in current-carrying nanostructures. We start from the analytical expression for the curl of these forces, and evaluate it for a point defect in a current-carrying system. We obtain a general definition of the capacity of electrical current flow to exert a nonconservative force, and thus do net work around closed paths, by a formal noninvasive test procedure. Second, we show that the gain in atomic kinetic energy over time, generated by nonconservative current-induced forces, is equivalent to the uncompensated stimulated emission of directional phonons. This connection with electron–phonon interactions quantifies explicitly the intuitive notion that nonconservative forces work by angular momentum transfer.

## Introduction

Electron–nuclear interactions lie at the heart of the transport properties of nanoscale conductors. Even in the limit of elastic (phonon-free) conduction, the nature and positions of nuclei in a nanojunction determine the external potential, experienced by current-carrying electrons, and, together with electron–electron interactions, determine the current–voltage spectrum of the system. Allowing nuclei to respond to current-induced forces introduces two additional elements: Current-driven displacements and Joule heating. Current-induced forces arise, fundamentally, through momentum transfer from the electron flow to nuclei, and are familiar from the field of electromigration [1]. An alternative, but fundamentally equivalent, way to think about them is as nonequilibrium corrections to interatomic bonding forces. In considering these forces, it is often convenient to adopt the Born–Oppenheimer approximation, adapted to the nonequilibrium conditions in a nanoconductor: We think of nuclei as heavy, slow classical particles, and imagine that, as nuclei move, electrons always remain in the steady state appropriate for the given set of instantaneous nuclear positions; we then calculate the force on a nucleus exerted by the mean electron density in the system (including appropriate Pulay corrections, if an incomplete electronic basis is used) [2–5]. Joule heating, on the other hand, is due to the finite mass of nuclei, and results from the recoil of nuclei in inelastic collisions with electrons [6–10]. The combined effect of the two is the driving force behind electromigration-type phenomena [2,6–7]: Current-induced forces modify atomic migration barriers; together with local heating, this results in thermally activated current-induced atomic rearrangements, or even failure.

Recently, a new and rather different aspect of current-induced forces has received attention: Their nonconservative character, and their resultant ability to do net work on individual atoms, or groups of atoms, around closed paths [1,11–14]. The practical consequences of this mechanism for sustained energy transfer from electrical current into atomic motion are only just starting to be explored. Two aspects of the effect that are of immediate interest are its capacity to drive an atomic-scale motor, and its possible potent role as a cause for dramatic mechanical failure [12–14]. Indeed, the notion that current can drive rotary motion, under appropriate conditions, is highly intuitive [15–16], and is increasingly being seen as a common, rather than rare, effect in nanoscale conductors [17]. The essential physics behind the nonconservative component of current-induced forces is that of a waterwheel driven by a flow [12]. For quantum-mechanical electron flow interacting with classical nuclei, the effect is quantified precisely by an analytical result for the curl of these forces [12,18]. Yet, there is a gap to be bridged between the formal result and the intuitive physics.

The aim of the present short paper is to bridge this gap, and extract explicitly the gas-flow picture of nonconservative current-induced forces. We then make a second connection, by showing that the work done by these forces around closed paths is equivalent to the uncompensated stimulated emission of directional phonons, characterised by the sign of their angular momentum. This second result will close the gap between the nonconservative effect and the more familiar fundamental physics of electron–phonon interactions.

## Results and Discussion

### The gas-flow picture

Under steady-state conditions, in the absence of phonons, the electronic properties of a nanoscale conductor are parametric functions of the classical nuclear positions. So too are the current-induced forces on the nuclei. The nonconservative component of these forces is characterised by the generalised curl expression [12,18]

[1]
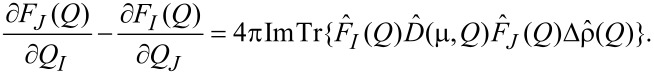


Here, and throughout the paper, we work in the small-bias limit. For our present purposes, we work with noninteracting electrons and zero magnetic fields.

In Equation 1, *Q* denotes a collection of generalised classical coordinates {*Q_I_*}; *F_I_*(*Q*) is the current-induced force on coordinate *Q_I_*; 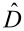
(μ,*Q*) = *δ*[μ − *Ĥ_e_*(*Q*)] is the operator for the electronic density of states, evaluated at the Fermi level μ; Δ
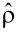
(*Q*) is the nonequilibrium part of the one-electron density matrix (that is, the difference between the steady-state current-carrying density matrix and the equilibrium density matrix); finally, 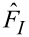
(*Q*) is the force operator

[2]
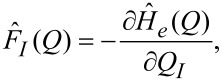


where *Ĥ_e_*(*Q*) is the one-electron Hamiltonian, as a parametric function of the classical degrees of freedom *Q*.

Equation 1 is intriguing but lacks transparency. We now probe its physical content as follows. We immerse a point defect, with position vector **R** = (*X,Y,Z*) and a scattering potential 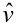
(**R**) = *Cδ*(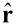
−**R**), in the electron flow. Here, **r** = (*x,y,z*) denotes the electron position. We shall use the defect to directly measure the ability of the flow to exert a nonconservative force.

We have

[3]
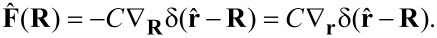


Below, we deliberately treat *C* as small and work to the lowest nontrivial order in this parameter. Taking the trace in Equation 1 in the continuum **r**-representation,

[4]
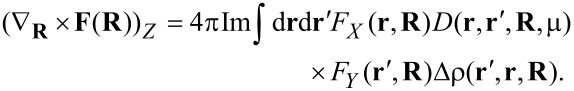


We also have

[5]
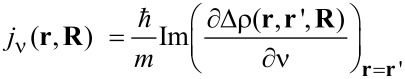


[6]
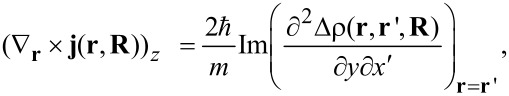


where ν = *x*,*y*,*z* and **j**(**r**,**R**) is the electron particle-current density.

Then, to lowest order in *C*, we obtain

[7]
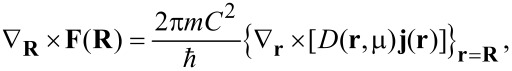


where *D*(**r**,μ) = *D*(**r**,**r**,μ) and **j**(**r**) are the local density of states at the Fermi level and the particle-current density *in the absence of the point scatterer*. Therefore, Equation 7 defines an *intrinsic property* of the current flow: Its “curl-generating” capacity. The point defect above serves as a noninvasive test particle that probes this property. One can picture situations in which this intrinsic curl vanishes but yet there still is a nonzero curl to higher order in the coupling between the scatterer and the electrons. One example of this interesting possibility would be an atom weakly bonded to a structure, where the weak bonding enables current to flow through the atom in the first place.

Equation 7 is completely general and makes no assumptions about the nature and structure of the conductor. We will now simplify it further as follows. We assume that all electronic properties vary slowly in space, and we attribute a local Fermi momentum, *p*(**r**), to the electrons. We now have a semiclassical gas flow in a, locally, jellium-like environment. Next, we observe that for jellium

[8]
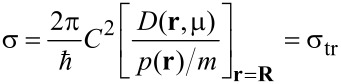


is the scattering cross section of the defect. In the assumed, locally free-electron-like medium, in 3D, to lowest order in *C*, σ is a constant (since *D*(**r**,μ) 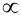 *p*(**r**)), and is equal also to the transport cross section, σ_tr_. Hence,

[9]
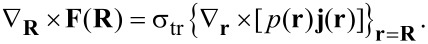


Thus, (the curl of) the force on the test particle is proportional to (the curl of) the local momentum flux of the flow, [*p*(**r**)**j**(**r**)], with a constant of proportionality σ_tr_. At this stage, quantum mechanics has all but disappeared from the problem: We have a classical interaction between a, generally, spatially nonuniform steady gas flow and an elastic scatterer in its path.

Finally, recognising

[10]
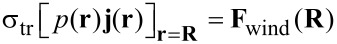


as the electron-wind force in electromigration, we have

[11]
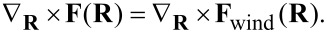


Therefore, we have shown from first principles that the point of departure, Equation 1, is an algebraic statement of Sorbello’s thought experiment [1] to prove that this force is, in general, a nonconservative force. We have shown, further, that the key quantity responsible for this property is the curl of the local electron momentum-current density.

### Nonconservative work as directional phonon emission

**Figure 1:**
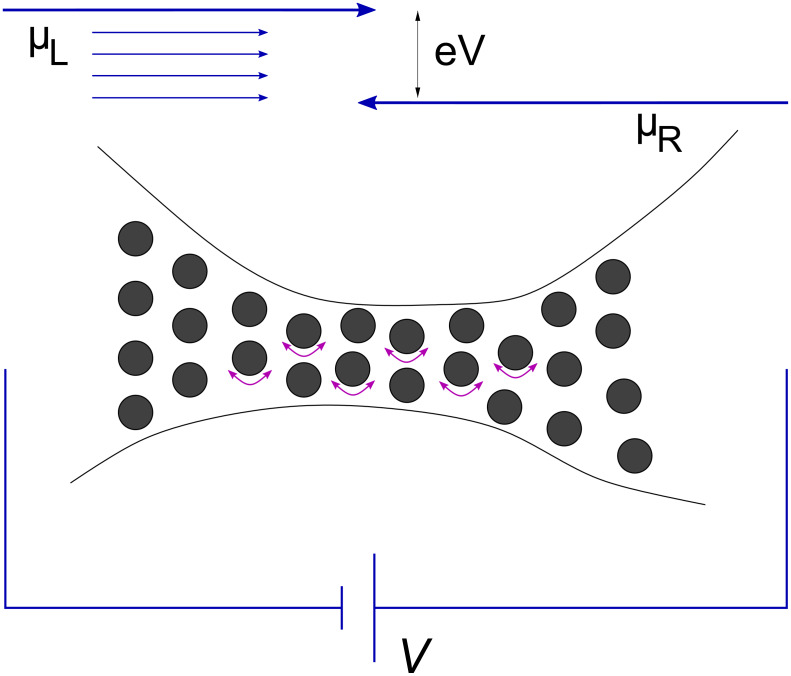
An electrode–junction–electrode system in the Landauer picture. The details are discussed in the text.

We will now relate the nonconservative current-induced forces on atoms to the intuitive idea of the waterwheel. To this end, we will show that the work done by these forces around closed paths corresponds exactly to the stimulated generation of directional phonons, characterised by their angular momentum. Consider a flux of electrons travelling through an elastic medium. Intuitively, we expect the flux to preferentially emit phonons with momentum parallel to the electron flow. This becomes evident, when we consider the setup in Figure 1.

A nanostructure is connected to two electrodes, each in turn connected to its own battery terminal serving as a particle reservoir. The left reservoir injects right-travelling electrons with electrochemical potential

[12]
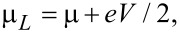


and the right reservoir injects left-travelling electrons with electrochemical potential

[13]
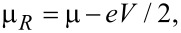


where *V* is the applied bias.

The current comes from the energy window between μ*_R_* and μ*_L_*, where there are partially-populated electron states. A right-travelling electron in this energy window can emit a right-travelling phonon, and scatter into a left-travelling state. But the reverse process is suppressed, due to the population imbalance between the two sets of electron states. Hence, we expect a directional preference of the emitted phonons. Since the rate of stimulated emission increases with growing phonon population, we might expect the process to have the ability to feed on itself. We will show below that this is the physical origin of the nonconservative current-induced forces.

Consider two independent generalised oscillator coordinates *X* and *Y*, with the same angular frequency, ω. Here, *X* and *Y* could be two individual atomic degrees of freedom (not necessarily of the same atom), or they could be collective normal modes. In all cases, we assume, as our starting point, that the modes *X* and *Y* describe *standing waves*. The Hamiltonian for the two oscillator degrees of freedom is

[14]
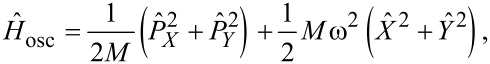


where *P_X_* and *P_Y_* are the corresponding canonical momenta, and *M* is a mass-like parameter.

We now form new modes, which we label by (+) and (−), with annihilation operators

[15]
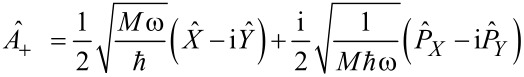


[16]
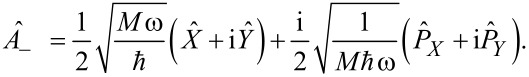


They obey

[17]
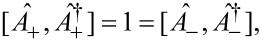


with all other commutators equal to zero. The inverse relations are

[18]
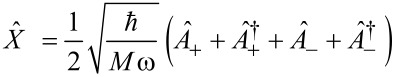


[19]
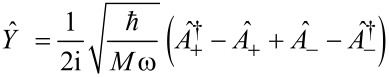


[20]
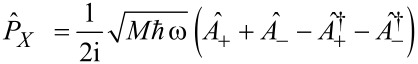


[21]
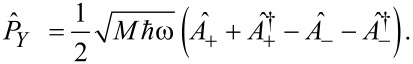


The new modes diagonalise the Hamiltonian as follows

[22]
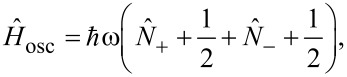


where

[23]
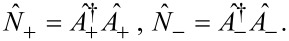


The physical significance of the new modes can be seen by considering the angular momentum

[24]
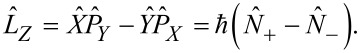


Thus, the “anticlockwise” mode (+) carries positive angular momentum (in the direction, perpendicular to the abstract *X*–*Y* plane) and the “clockwise” mode (−) carries negative angular momentum. By coupling these two directional phonon modes to electrons, we will see that the electron current pumps energy into one, while damping the other.

The coupling between electrons and phonons is described by scattering theory. The unperturbed, phonon-free state of the current-carrying electrons is that of the usual Landauer picture [5]. In this picture, electrons in the phonon-free steady state are described by two sets of stationary one-electron Lippmann–Schwinger scattering states. One set, {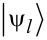} with energies {*E_l_*}, originate from the left electrode, and are scattered elastically at the junction, with partial backscattering into the left electrode and partial transmission into the right electrode; the other set, {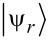} with energies {*E_r_*}, originate from the right electrode, with partial backscattering into the right electrode and partial transmission to the left. In the absence of bound states (which we assume here), the two form a complete orthonormal set. The left reservoir populates states *l*, in a grand-canonical ensemble with electrochemical potential μ*_L_* (and a chosen electronic temperature); the right reservoir populates states *r* with electrochemical potential μ*_R_*. It is convenient to define the density of states operators [5]

[25]
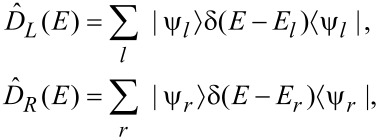


with

[26]
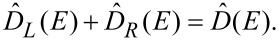


Here, we include spin in 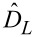
(*E*) and 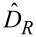
(*E*). From Equation 12, Equation 13 and Equation 25, the nonequilibrium part of the one-electron density matrix, in the linear bias regime, is given by

[27]
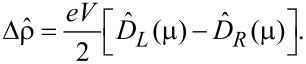


The system of two oscillators, we describe by an unperturbed density matrix that commutes with 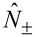. Electrons and oscillators are then coupled by an interaction of the generic form

[28]
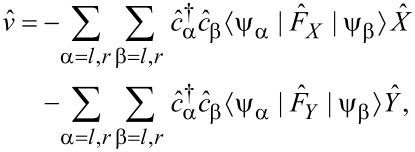


where 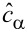 and 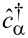 are fermionic annihilation and creation operators. Then, for the resultant rates of change of the occupancies, *N*_±_, of the anticlockwise and clockwise modes, to lowest order in 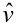, we obtain

[29]
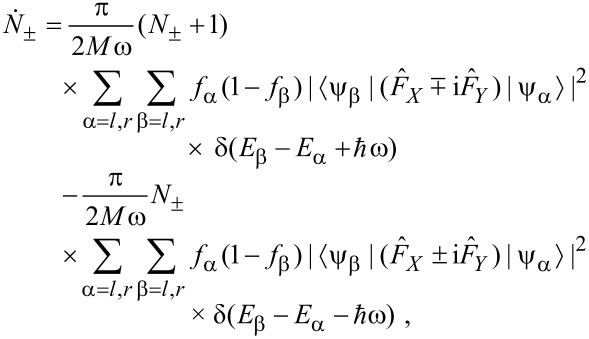


where *f_l_* = *f_L_*(*E_l_*) and *f_r_* = *f_R_*(*E_r_*) are the Fermi–Dirac distributions for electrons originating from the two respective reservoirs.

We now deliberately suppress the spontaneous phonon emission. Formally, we work in the classical limit and set (*N*_±_ + 1) ≈ *N*_±_. Then, counting all possibilities for α and β above and observing the selection rules, setting the electronic temperature to zero, and ignoring variations in the electronic properties over energies in the region of 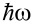 or *eV*, we get

[30]
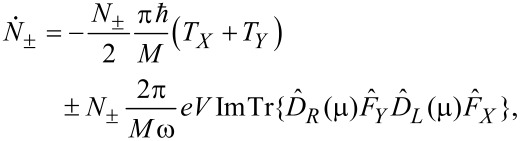


where

[31]
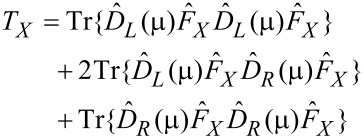


[32]
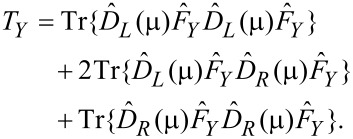


This is our final result. Equation 30 displays precisely the picture from [12]. Mode (+) experiences a damped driven motion. The damping (first term) is due to the ordinary electronic friction experienced by the two independent modes *X* and *Y* (each of which carries half of the energy of mode (+)). This friction is due to phonon absorption by electrons, and is present even at zero current. The driving term (the second term) comes solely from the current. It anti-drives mode (−).

To extract explicitly the curl of the effective driving force acting on the 2D oscillator, for the rate at which this force does work on mode (+) we write

[33]
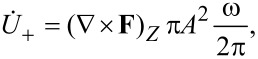


where *U*_+_ = *N*_+_ 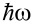 = *M*ω^2^*A*^2^ is the energy of the mode and *A*^2^ is the mean square displacement in the *X*–*Y* plane. Comparison with the second term in Equation 30 gives

[34]
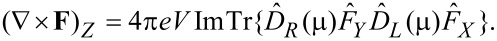


In view of Equation 26 and Equation 27, this is equivalent to Equation 1 [13]. The difference is that in [12,18] this result was derived in the mixed quantum-classical framework of Ehrenfest dynamics. Herein, it has been obtained formally exactly from quantum-mechanical electron–phonon coupling: The work done by nonconservative forces around a closed path is equivalent to the stimulated emission of directional, angular-momentum-carrying phonons. The opposite sign of the effect for modes (+) and (−) originates from the directionality, introduced by the current, that breaks the symmetry between clockwise and anticlockwise atomic motion in the *X*–*Y* plane [17]. The averaging over classical trajectories, implicit in the construction of the unperturbed phonon density matrix in the present calculation, eliminates certain additional forces that become apparent, for example, in the treatments of references [13–14]. These additional forces and their effects present an interesting avenue for further work [19].

## Conclusion

We have taken the analytical result for the curl of current-induced forces, derived in references [12,18], and we have related it to two physically transparent ideas. One is the electron-wind force on a test particle, determined by the local electron momentum flux. The second is the uncompensated stimulated emission of travelling phonons. The fact that the nonconservative effect is related to stimulated (as opposed to spontaneous) emission explains the remarkable, and practically very useful, earlier finding that the nonconservative dynamics of atoms under current can be captured already at the level of Ehrenfest dynamics. (Ehrenfest dynamics suppresses spontaneous transitions but retains stimulated transitions.) However, at that level, the underlying physics of the effect remains somewhat obscured by the mixed framework. Starting from the present internally consistent picture, we see explicitly that this novel and interesting effect is like a current-driven waterwheel, which works by angular-momentum transfer from the electron flow (albeit possibly in an abstract sense, depending on what *X* and *Y* denote).

Superficially, Equation 30 resembles ordinary Joule heating. Indeed, it does constitute a form of *directional* heating. But there is a key difference. *Standing*, bound phonon modes can equilibrate with the current-carrying electrons, at an effective phonon population set by the bias [20]. Equation 30 shows qualitatively different behaviour. If the second term is positive, then, once the bias *V* is large enough for term 2 to dominate term 1, the Equation predicts an exponential growth of the energy of the given *travelling* mode, without equilibration. This is the waterwheel effect of reference [12]. Of course, in reality this increase cannot continue indefinitely, and the possible outcomes form the subject of ongoing research. Pertinent questions are concerned with the effects of anharmonicity [12], the possible eventual failure of the device [13], and the possible current-induced corrections, under appropriate conditions, to the conservative part of the harmonic potential (which could lift the degeneracy of the modes *X* and *Y* that form the “waterwheel”).

It is hoped that nonconservative forces, and the underlying mechanism of uncompensated directional phonon generation under current, will be useful in tackling not only problems in nanotechnology but also in neighbouring areas, such as the behaviour of bulk metals under large current densities.
